# Combinatory Therapy Antimicrobial Peptide-Antibiotic to Minimize the Ongoing Rise of Resistance

**DOI:** 10.3389/fmicb.2019.01703

**Published:** 2019-08-09

**Authors:** Luis R. Pizzolato-Cezar, Nancy M. Okuda-Shinagawa, M. Teresa Machini

**Affiliations:** Peptide Chemistry Laboratory, Department of Biochemistry, University of São Paulo, São Paulo, Brazil

**Keywords:** antibiotic-resistant organisms, multidrug-resistant organisms, microbial infections, infection diseases treatment, biofilm-forming organisms, antimicrobial peptides

## The Antibiotic Resistance Crisis

Antibiotics are cytotoxic or cytostatic compounds, very effective, harmful and specific against pathogenic microorganisms that have saved millions of lives and increased human life expectancy and quality (Zaman et al., 2017). Nevertheless, we have almost reached a post-antibiotic era, where even simple infections have become untreatable due to the remarkable rise of resistance (Chaudhary, 2016).

Antibiotic resistance is the ability of microorganisms to withstand the effect of medicines. Although an inevitable natural phenomenon, the abusive use of antibiotics has provided constant selection pressure and accelerated the emergence of highly resistant strains (Richardson, 2017). It is perhaps not intuitive, but it is estimated that the vast majority of all antibiotics produced is used improperly in the food-animal sector to promote rapid growth and prevent infectious diseases, rather than in human medicine (Landers et al., 2012). This article focuses on the main aspects of the combinatory therapy antimicrobial peptide (AMP)-antibiotic to treat infectious diseases.

## Combinatory Therapy AMP-antibiotic

Antibiotic resistance management is an attempt to decrease the resistance rate. It demands both the limitation of antibiotic use and the application of more efficient infection therapies. Since antibiotic exposure time correlates with the development of resistance, effective therapies should include drugs with rapid death kinetics and broad spectra of action. Such requirements are mainly found in combinatory therapies, which in contrast to monotherapies, simultaneously employs different drugs to treat a particular disease. Combining different drugs mainly leads to synergism or antagonism. In a synergistic response, the combination has a considerably stronger effect than single drugs would, more than just an additive effect. It meaningfully improves clinical outcomes and decreases the probability of resistance evolution since it is unlikely that a pathogen simultaneously develops resistance to multiple antibiotics (Xu et al., 2018). Correctly choosing the combination cocktail is a crucial step and AMPs have been increasingly recognized as a promising class of compounds to be used in combination with classic antibiotics for the treatment of various infections (Lewies et al., 2018).

AMPs are composed of amino acids, typically 5–50 residues, and produced by all classes of multicellular organisms as an essential part of the innate immune response. Usually, they target a broad range of essential metabolic processes of bacterial and fungal cells. The main characteristic of most AMPs is its positive net charge, which allows for the interaction with negatively charged components of the cell wall and plasma membrane. Following interaction, amphipathic AMPs insert into the membrane, a process driven by the presence of hydrophobic amino acids. Subsequent membrane disruption occurs by a variety of mechanisms, leading to loss of its integrity and, ultimately, cell death (Carvalho et al., 2015). In addition to membrane-lytic activities, AMPs also exert intracellular inhibitory activity by interfering with diverse essential processes as protein biosynthesis, cell division, cell-wall biosynthesis and nucleic acid metabolism. Furthermore, in complement to antimicrobial activities, AMPs also modulate the immune response stimulating cytokine production, acting as chemokines, and promoting wound healing (Bechinger and Gorr, 2017).

Presumably, the crucial advantage of AMPs is that they are described as less prone to induce resistance as most have multiple targets and rarely interact with a specific receptor. Among those with a single target, most act on the membrane where resistance evolution is more unlikely to occur (Sierra et al., 2017). However, in exceptional cases involving specific protein interactions, the possibility of genetic mutation and resistance development is a significant event but unlikely in combinatory therapy AMP-antibiotic due to the magnitude of different targets involved. Moreover, this rare event can be overcome by slight structural modifications that is easy and rapid for AMPs due to the tremendous progress made in solid phase peptide synthesis. Such advance resulted from the availability of low-cost high-quality building blocks and coupling reagents, establishment of efficient approaches and protocols to speed up peptide assembly, and the development of fully automated synthesizers (Mijalis et al., 2017).

In the last few years, several studies have demonstrated the benefits and advantages of combinatory AMP-antibiotic therapy, which include the successful elimination of multidrug-resistant (MDR) and biofilm-forming organisms, a significant lower outcome of resistance development, reduction of single doses and a decrease in side effects (Lewies et al., 2018). Perhaps one of the leading causes of resistance development is the low microbial cell membrane permeability to antibiotics (especially the outer membrane of gram-negative bacteria that is primarily composed of polyanionic lipopolysaccharides) and since most AMPs act on membranes, perturbing their structures, the combinatory therapy AMP-antibiotic arises as an efficient tool to increase antibiotic bioavailability (Li et al., 2017). Indeed, recent studies have shown that in particular cationic AMPs, such as LL-37, piperacillin, buforin II, ceprocin P1, indolicidin, nisin, and magainin II, are remarkably effective in combination with antibiotics like polymyxin E, piperacillin, azithromycin, daptomycin, linezolid, and clarithromycin to enhance antibiotic bioavailability against highly multidrug-resistant gram-negative and methicillin-resistant *S. aureus* (MRSA) pathogens (Giacometti et al., 2000; Mataraci and Dosler, 2012; Lin et al., 2015). These studies are of enormous importance as increasing bioavailability reduces the required antibiotic concentration and, consequently, the probability of resistance development.

More than to enhance oral bioavailability, the strong membrane permeabilization capacity of AMPs can directly kill even dormant biofilm-forming cells in combination with classical antibiotics. An example demonstrating the efficacy of AMP-antibiotic therapy to remove biofilm is the treatment of *Pseudomonas aeruginosa* (*P. aeuruginosa*) with carbapenems. Such antibiotics belong to the class of broad-spectrum antimicrobials routinely used for the treatment of infections caused by multidrug-resistant *P. aeuruginosa* that leads to chronic diseases. Recently, a novel synthetic cyclolipopeptide analog of polymyxin (AMP38) was tested in combination with carbapenems, and the synergistic effect was observed to cause the killing of biofilm-forming and carbapenem-resistant *P. aeruginosa* (Rudilla et al., 2016). Since biofilm represents an enormous obstacle in antibiotic-therapy, this area has recently received increased attention from the scientific community, given the high number of reports demonstrating the benefits of combination AMP-antibiotics for the treatment of biofilm-forming organisms (Reffuveille et al., 2014; Ribeiro et al., 2015; Grassi et al., 2017).

Besides affecting membrane integrity, some AMPs also have intracellular targets. For instance, arenicin-1 in combination with a broad spectrum of antibiotics increases drug bioavailability and promotes oxidative stress by depletion of NADH (Choi and Lee, 2012). Similarly, buforin II was primarily shown to act on the membrane, but as it was demonstrated later, it also interacts with DNA, interrupting DNA and RNA metabolisms (Sim et al., 2017). It is important to note that since most AMPs have multiple cell targets, their mechanisms of action are strictly dependent on the concentration. For instance, studies conducted with pleurocidin has shown that, at its lowest inhibitory concentrations, this is less able to damage cell membranes but capable of inhibiting macromolecular synthesis (Patrzykat et al., 2002). Indeed, typically, AMPs cause membrane lysis at high concentrations and no-membrane lysis at low concentrations (Cudic and Otvos, 2002).

It is also essential to emphasize that the combinatory therapy AMP-antibiotic is effective to treat diseases caused by MDR organisms as for such proposes the essential requirement of the drug cocktail is to have components with different killing mechanisms. For example, the combination of the antimicrobial peptide DP7 with azithromycin or vancomycin was shown to eradicate some antibiotic-resistant bacteria like *Staphylococcus aureus* (*S. aureus*), *P. aeruginosa*, and *Escherichia coli* (*E. coli*) (Wu et al., 2017). Analogously, the AMP SET-M33 was extremely effective against a set of gram-negative MDR organisms as *Klebsiella pneumoniae* (*K. pneumoniae*), *P. aeruginosa* and *Acinetobacter baumannii* (*A. baumannii*), especially in combination with rifampin (Pollini et al., 2017). Even in cases where the AMP alone has just a moderate antimicrobial activity, its combination with antibiotics was effective against MDR organisms. Indeed, as recently shown, a combination of ASU014, a bivalent branched peptide with moderate activity against *S. aureus*, with oxacillin was also very efficient against MRSA. The synergism between both meaningfully improved the killing effect as compared to single drugs, so that lower peptide concentrations and sub-MIC doses of the antibiotic were required for the complete eradication of the pathogen (Lainson et al., 2017).

Closely related to resistance is persistence, a phenomenon in which microorganisms become insensible toward lethal antibiotic doses not due to genetic acquired modifications, but by entering in a dormant and drug-tolerant state. This state is transient and lasts as long as the stress condition endures. Consequently, persistence is directly related to chronic and recurrent infectious diseases. The mediation of persistence occurs by the signaling molecule ppGpp in response to environmental stress as the presence of antibiotics (Pollini et al., 2017). A recent study of the synergistic effect between a broad set of AMPs and antibiotics like ciprofloxacin, meropenem, erythromycin, and vancomycin for treating infections caused by clinical hard-to-treat pathogens, including all ESKAPE (*Enterococcus faecium, S. aureus, K. pneumoniae, A. baumannii, P. aeruginosa, Enterobacter cloacae*) pathogens, revealed the ability of AMPs to elicit degradation of ppGpp, avoiding the entry in an energy-starved state. This is a significant finding as potentially all microorganisms react to antibiotic treatment mediating persistence, and the fact that some AMPs can prevent it opens new doors for the development of alternative therapies that effectively decrease the resistance rate (Pletzer et al., 2018).

In addition to all those benefits, some AMPs can confer protection by acting as potent immune regulators by means of chemokine, inhibiting pro-inflammatory cytokine production and modulating the response of the adaptive immune response via the regulation of T cells (Diamond et al., 2009). In this regard, LL-37 is probably the most tested AMP in combinatory therapy. A study performed to assess the antibacterial activity of amoxicillin with clavulanic acid and amikacin against different clinical isolates of *S. aureus* revealed that the killing effect of the antibiotic alone was strongly potentiated by the addition of synthetic LL-37 (Leszczyńska et al., 2010). Similarly, the anti-tuberculosis antibiotics isoniazid and rifampicin were shown to clear *Mycobacterium tuberculosis* (*M. tuberculosis*) from infected lungs, liver, and spleen, more efficiently in combination with the human neutrophil peptide (HNP)-1 in comparison to when they were employed alone (Kalita et al., 2004).

In summary, the benefits of AMPs associated with the potency of conventional antibiotics in combinatory therapy can very efficiently favor the resolution of infections caused by MDR and biofilm forming microorganisms, enhances the natural immune response and decreases the likelihood of resistance.

## Barriers for the Therapeutic Use of AMPs

In clinical therapy, the most desirable route of drug administration is orally due to the relatively low cost of production and patient compliance (Zhu et al., 2017). However, before any drug reaches the bloodstream, and consequently its target, it will typically face many obstacles that include the mouth environment and the harsh gastric tract containing digestive enzymes, highly viscose mucosal layers, epithelial cells preventing the direct contact with the capillary and tight junctions between the epithelial, blocking the paracellular passage. For peptides, all those barriers restrict their ease of administration due to their low cell membrane permeability and limited stability toward proteolysis (Lewis and Richard, 2015). In fact, according to THPdb (http://crdd.osdd.net/raghava/thpdb/), a database for therapeutic peptides and proteins, only 4 % of all approved therapeutic peptides and proteins are administered orally. The following sections focus on the stability issue and the low membrane permeability of peptides in general and present some successful strategies that overcome the practical limitations of peptides as orally administered drugs.

Except cyclic and D-amino acid composed AMPs, the majority is linear and formed by natural L-amino acids. Thus, they are similar to food peptide/proteins and substrates of several digestive enzymes. Nevertheless, most proteases exclusively recognize the 20 natural L-amino acids and, consequently, the stability of many AMPs can be enhanced by the addition of chemical modifications, replacement of L-amino acids by their D-isomers (Remuzgo et al., 2014) and chain cyclization. In contrast to conventional antibiotics, AMPs tolerate more modifications while maintaining their activity. As already discussed, most AMPs form disruptive pores in the membrane, an event that is primarily driven by physical properties like net charge rather than by amino acids conformation. Thus, changing L-amino acids to the corresponding D-isomers usually does not impair AMP activity. Even in cases involving specific receptor-AMP interaction, replacements and modifications might not necessarily impair AMP action. Unlike classic antibiotics, the interaction surface AMP-receptor is usually more extensive, and the replacement of natural amino acids by non-natural analogs is less pronounced and, in some cases, can even improve the affinity. A study comparing the impact of many modifications has revealed that the addition of alpha-methyl amino acids and D-analogs confers to the peptide the most pronounced protection from proteolysis without activity loss (Werner et al., 2016).

In addition to the stability problem, oral delivery of AMPs is also challenging due to their poor cell membrane permeability. Once orally administered, AMPs should cross the gastrointestinal epithelium to reach the bloodstream. However, this is not so simple for hydrophilic molecules exceeding 700 Da (Fosgerau and Hoffmann, 2015). Nonetheless, it has been shown that the successful transport of different molecules like proteins, peptides or DNA across the biological membrane could be achieved by the simultaneous addition or fusion of the molecule of interest with a class of transcellular enhancers known as cell penetrating peptides (CPPs). Such molecules are short peptides able to cross cellular membranes via an energy-dependent or independent mechanism. Their chemical nature is diverse, but most CPPs are positively charged; a primary or secondary amphipathic character can also be implicated but is not strictly required for internalization. In fact, it has been reported that even octa-arginine can mediate cellular uptake when co-administered or in conjugation with a cargo molecule (Dinca et al., 2016). Conjugation of CPPs with clinically relevant molecules was reported. Examples include the combination of lipo-polyarginine with insulin that was shown to enhance the transport through Caco-2/HT-29 cells almost two-fold (Garcia et al., 2018). Moreover, the conjugation or co-administration of TAT and polynonaarginin with the parathyroid hormone has sharply increased the transport through Caco-2 cells (Kristensen et al., 2015). However, in cases where the AMP should act in the gastrointestinal tract, low bioavailability is desired as the MIC value is more likely to be reached by lower doses. Thus, only proteolytic stability remains a possible issue. For instance, surotomycin is a cyclic lipopeptide antibiotic active against *Clostridium difficile* (*C. difficile*), and as clinical evidence has shown, its low oral bioavailability allows the gastrointestinal tract concentrations to considerably exceed its MIC for the pathogen (Knight-Connoni et al., 2016).

## Perspectives

The combinatory therapy AMP-Antibiotic has increasingly attracted attention within contemporary studies due to its diverse benefits. The number of combination studies involving AMP-antibiotic has therefore been exponentially growing over the last few years (Jorge et al., 2017). However, the peptide permeability/stability problem remains the main obstacle for the use of peptides in clinical therapy. Currently, no straightforward solution is available, but great efforts have been made to develop targeted AMPs and to turn peptides into more appropriate drugs for oral use. In addition, the standardization of methods used to determine the synergism between AMPs and antibiotics, their interactions and the creation of antimicrobial combination networks has been facilitating combinatory studies (García-Fuente et al., 2018; Pemovska et al., 2018). Given the promising results obtained so far, the trend shows that the appeal of using combinatory therapy AMP-antibiotic will become even greater. It could represent the beginning of a modern and efficient era in the battle against infectious diseases.

## Author Contributions

LP-C and NO-S are doctoral students who contributed to the preparation of the article. All authors contributed equally to this work.

### Conflict of Interest Statement

The authors declare that the research was conducted in the absence of any commercial or financial relationships that could be construed as a potential conflict of interest.
